# Genomic diversity of pathogenic *Escherichia coli *of the EHEC 2 clonal complex

**DOI:** 10.1186/1471-2164-10-296

**Published:** 2009-07-03

**Authors:** Galeb S Abu-Ali, David W Lacher, Lukas M Wick, Weihong Qi, Thomas S Whittam

**Affiliations:** 1 Microbial Evolution Laboratory, National Food Safety & Toxicology Center, 165 Food Safety & Toxicology Building, Michigan State University, East Lansing, Michigan 48824, USA; 2 Division of Molecular Biology, Center for Food Safety and Applied Nutrition, U.S. Food and Drug Administration, Laurel, Maryland 20708, USA; 3 Biosynth AG, Rietlistrasse 4, 9422 Staad, Switzerland; 4 Functional Genomics Center Zurich, University of Zurich and Swiss Federal Institute of Technology Zurich, Winterthurerstrasse 190, 8057 Zurich, Switzerland

## Abstract

**Background:**

Evolutionary analyses of enterohemorrhagic *Escherichia coli *(EHEC) have identified two distantly related clonal groups: EHEC 1, including serotype O157:H7 and its inferred ancestor O55:H7; and EHEC 2, comprised of several serogroups (O26, O111, O118, etc.). These two clonal groups differ in their virulence and global distribution. Although several fully annotated genomic sequences exist for strains of serotype O157:H7, much less is known about the genomic composition of EHEC 2. In this study, we analyzed a set of 24 clinical EHEC 2 strains representing serotypes O26:H11, O111:H8/H11, O118:H16, O153:H11 and O15:H11 from humans and animals by comparative genomic hybridization (CGH) on an oligoarray based on the O157:H7 Sakai genome.

**Results:**

Backbone genes, defined as genes shared by Sakai and K-12, were highly conserved in EHEC 2. The proportion of Sakai phage genes in EHEC 2 was substantially greater than that of Sakai-specific bacterial (non-phage) genes. This proportion was inverted in O55:H7, reiterating that a subset of Sakai bacterial genes is specific to EHEC 1. Split decomposition analysis of gene content revealed that O111:H8 was more genetically uniform and distinct from other EHEC 2 strains, with respect to the Sakai O157:H7 gene distribution. Serotype O26:H11 was the most heterogeneous EHEC 2 subpopulation, comprised of strains with the highest as well as the lowest levels of Sakai gene content conservation. Of the 979 parsimoniously informative genes, 15% were found to be compatible and their distribution in EHEC 2 clustered O111:H8 and O118:H16 strains by serotype. CGH data suggested divergence of the LEE island from the LEE1 to the LEE4 operon, and also between animal and human isolates irrespective of serotype. No correlation was found between gene contents and geographic locations of EHEC 2 strains.

**Conclusion:**

The gene content variation of phage-related genes in EHEC 2 strains supports the hypothesis that extensive modular shuffling of mobile DNA elements has occurred among EHEC strains. These results suggest that EHEC 2 is a multiform pathogenic clonal complex, characterized by substantial intra-serotype genetic variation. The heterogeneous distribution of mobile elements has impacted the diversification of O26:H11 more than other EHEC 2 serotypes.

## Background

Enterohemorrhagic *Escherichia coli *(EHEC), the intersection of Shiga toxin producing *E. coli *(STEC) and attaching and effacing *E. coli *(AEEC), comprise a group of pathogenic *E. coli *that cause a variety of human and animal illnesses ranging from diarrhea to hemorrhagic colitis (HC), and the multifactorial hemolytic uremic syndrome (HUS) [1]. Intimate adherence to the intestinal epithelium resulting in characteristic attaching and effacing (A/E) lesions, and the destruction of capillary walls via production of phage borne Shiga toxins (Stx 1, 2, and variants) are hallmarks of EHEC pathogenesis. A/E lesion formation is dependent upon a type three secretion system (TTSS), which is encoded on the laterally acquired locus of enterocyte effacement (LEE) [2].

*E. coli *O157:H7 is the dominant EHEC serotype in the United States, Argentina, Great Britain, and Japan [3,4]. However, multiple reports have shown that other EHEC, including serogroups O26, O111, O103, and O118, frequently cause sporadic cases of human illness [5-12], and have been implicated in numerous outbreaks [13-17]. In Australia and parts of Europe, infections with serogroups O26 and O111 are prevailing while the incidence of O157:H7-associated disease appears to be declining [18-21]. In contrast to *E. coli *O157:H7, EHEC serogroups O26, O111, O118, O103, and O5 are commonly linked to outbreaks and sporadic cases of calf diarrhea (scours) and HC [22-28], which has been validated from experimental infections in calves [29-32]. In Germany and Belgium, for example, EHEC O118 is the most prevalent type of STEC associated with diarrhea in calves [33], with evidence for zoonotic transmission [8,34].

Phylogenetic analyses of conserved metabolic genes have revealed some of the basis for the variation among EHEC strains. Multilocus enzyme electrophoresis [35] and partial sequencing of 13 housekeeping genes [36] classified EHEC into two distantly related clonal groups: EHEC 1 includes serotype O157:H7 and its inferred ancestor O55:H7, whereas EHEC 2 includes numerous serogroups (e.g., O26, O111, O118). The key virulence factors shared between EHEC 1 and EHEC 2 clonal complexes were postulated to have been introduced through multiple and parallel acquisitions of mobile elements [37]. A comparison of *E. coli *O157:H7 genomes has also revealed the extent and significant impact of horizontal transfer on the evolution of virulence [38,39]. Furthermore, array comparative genomic hybridizations (CGH) have shown that the divergence in gene content among closely related O157 strains is ~140 times greater than the divergence at the nucleotide sequence level [40]. Although recent evidence indicates the emergence of highly virulent lineages among non-O157 EHEC, notably the O26 serogroup [19,41], little is known about the gene content, genetic diversity and evolution of virulence in members of the EHEC 2 group.

The function of ancillary virulence determinants is somewhat characterized in O157:H7 [2,42], however, the relevance as well as the distribution of these factors in EHEC 2 is not clear. To systematically investigate the gene content variations within the EHEC 2 clonal group. we analyzed a set of 24 clinical EHEC 2 strains representing serotypes O26:H11, O111:H8/H11, O118:H16, O153:H11 and O15:H11 from humans and animals using array-based CGH. Because there are no EHEC 2 genome sequences available, a multi-genome spotted oligoarray containing probes for 5,978 ORFs from O157:H7 Sakai, O157:H7 EDL933, and K-12 MG1655 was used to examine the distribution of these *E. coli *genes in our collection of EHEC 2 strains. The findings of this study shed light on the diversification of horizontally acquired elements in a group of pathogens that represent recent evolutionary branches of EHEC clonal groups.

## Results

### Sequence types (STs) and *stx *profiles of EHEC 2 strains

**Figure 1:**
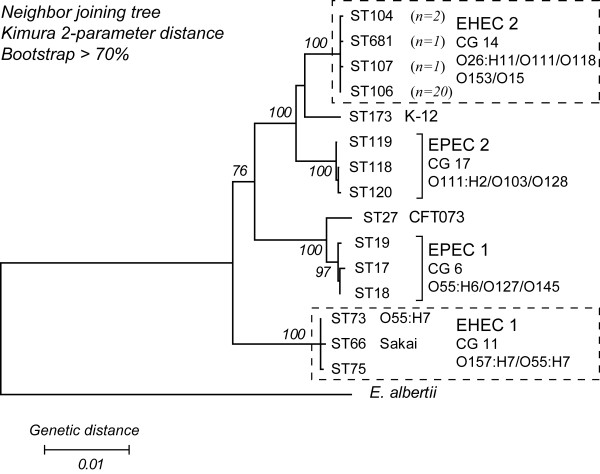
**Phylogenetic relationships of EHEC and EPEC sequence types**. The sequence types (STs) of EHEC 2 belong to a clonal group (CG 14), which is more closely related to EPEC 2 (CG 17), than EHEC 1 STs (CG 11). The phylogenetic tree was constructed using the Neighbor-joining algorithm based on the Kimura 2-parameter distance matrix of nucleotide substitution. Bootstrap confidence values were based on 1000 replicates. Only those higher than 70% are shown.

Phylogenetic analyses of multi locus sequence typing (MLST) data grouped the 24 EHEC 2 strains (Table 1) into four STs. The most common was ST 106, which was found in 20 strains, while the remaining three STs each differed from ST 106 by a single nucleotide polymorphism (SNP) in almost 4,000 bp of the concatenated MLST sequence. MLST data revealed a lack of nucleotide sequence diversity in house keeping genes among these EHEC 2 strains. The neighbor-joining phylogeny based on concatenated MLST allelic sequences grouped the EHEC 2 strains into a distinct cluster, with 100% bootstrap support, which was more closely related to the EPEC 2 group (100% bootstrap support) than to members of EHEC 1 (Figure 1). Most of these EHEC 2 strains (*n *= 17) were PCR positive for only *stx1*, whereas four strains had both *stx1 *and *stx2*, and three strains were negative for both *stx *genes (Table 1).

**Table 1:** Properties of strains used in this study sorted by serotype.

Strain^a^	Serotype^b^	Host	Clinical^c^	Location	Date^d^	*stx*	ST^e^	Source^f^, Reference
DEC 9f	O26: [h11]	Human	diarrhea	USA, S. D.	1974	-	106	CDC, [80]
DEC 10e	O26:H11	Calf	scours	USA, S. D.	1989	1	106	Francis, D., [81]
F5863	O26:H11	Human	diarrhea	USA, Nebr.	1998	1	106	Fey, P., [6]
97–3250	O26:H11	Human	HUS	USA, Idaho	1997	1,2	104	O'Brien, [82]
413/89-1	O26: [h11]	Calf	diarrhea	Germany	1998	1	106	Wieler, L., [83]
DA-22	O26: [h11]	Human	diarrhea	USA, D.C.	1999	1	106	Acheson, D. W.
03-ST-296	O26:H11	Human	b. d.	USA, Mich.	2003	1	106	MDCH, [84]
CB 7505	O26:H11	Calf	no data	Germany	1998	1	106	Beutin, L., [85]
DEC 8c	O111: [h11]	Calf	scours	USA, S. D.	1986	1	107	Francis, D., [80]
DEC 8d	O111:H11	Human	diarrhea	Cuba	1953	-	106	Orskov, F., [86]
C408	O111: [h8]	Calf	diarrhea	Scotland	1993	1	106	Hart, C. A., [87]
BCL71	O111: [h8]	Calf	diarrhea	USA, Calif.	1993	1,2	106	Love, B.C.
ML178190	O111: [h8]	Human	diarrhea	USA, Nebr.	1998	1,2	106	Fey, P., [6]
W29104	O111:H8	Human	diarrhea	USA, Nebr.	1998	1,2	106	Fey, P., [6]
EK34	O111: [h8]	Human	diarrhea	USA, Wash.	1999	1	106	Tarr, P., [88]
EK35	O111:H8	Human	diarrhea	USA, Wash.	2001	1	106	Tarr, P., [88]
RW2030	O118: [h16]	Calf	diarrhea	Germany	1994	1	106	Wieler, L., [33]
RW1302	O118: [h16]	Calf	diarrhea	Germany	1994	1	106	Wieler, L., [33]
666/89	O118:H16	Calf	diarrhea	Germany	1989	1	106	Wieler, L., [33]
05482	O118:H16	Human	HUS	Germany	1996	1	106	Beutin, L., [89]
EK36	O118:H16	Human	diarrhea	USA, Wash.	2001	1	106	Tarr, P., [88]
EK37	O118:H16	Human	diarrhea	USA, Wash.	2000	1	106	Tarr, P., [88]
RDEC-1	O15: [h11]	Rabbit	diarrhea	USA, S.C.	1970s	-	681	ECRC, [81]
02–3751	O153: [h11]	Rabbit	HUS	USA, Mass.	2002	1	104	Fox, J., [90]
97–3256	O55:H7	Human	diarrhea	USA, Mich.	1997	2	73	O'Brien, [82]

a. designations assigned to strains deposited in the STEC Reference Center

b. [h] – flagellar allele determined by *fliC *gene sequencing; H – expression of flagellar type confirmed by reaction to antisera. To avoid confusion in text, flagellar type will be denoted as H, regardless whether it was determined by sequencing or serologic typing.

c. b. d. – bloody diarrhea; HUS – hemolytic uremic syndrome; scours – neonatal calf diarrhea.

d. Year of isolation.

e. ST – sequence type based on MLST of 7 housekeeping genes (*aspC*, *clpX*, *fadD*, *icdA*, *lysP*, *mdh*, and *uidA*).

f. MDCH – Michigan Dept. of Community Health.

### Gene content of EHEC 2 strains

Binary classification of genes as present or divergent/absent, inferred by GACK analyses of the CGH data, was used to determine the gene content of all 24 EHEC 2 strains (Table 2) and of each individual strain (Table 3). Because all CGH experiments were performed with Sakai as the reference strain, our analyses focused on probes targeting genes present in the Sakai genome. The oligo probes were classified to represent backbone genes (shared by Sakai and K-12), and Sakai-specific genes (note that the term "Sakai-specific" is used here only in comparison to K-12). The Sakai-specific genes were further classified in Sakai phage genes (phage-related genes present in Sakai but absent in K-12) and Sakai bacterial genes (non-phage-related genes present in Sakai but absent in K-12) [38]. Of the 3,696 backbone genes, 80.9% were shared by all EHEC 2 strains, whereas only 5.8% of the Sakai phage genes (*n *= 814) and 6.5% of the Sakai bacterial genes (*n *= 434) were found in every tested EHEC 2 strain. While 84.7% of the Sakai phage genes were found in at least one of the 24 EHEC 2 strains, a whole 53% of the Sakai bacterial genes were not found in any of the these strains (Table 2).

**Table 2:** Percentage of Sakai genes that are present, divergent/absent or variably absent or present (VAP) in all 24 EHEC 2 strains.

	Backbone (shared with K-12) *n *= 3696	Sakai-specific	
		
		phage-related *n *= 814	Bacterial *n *= 434
Present	80.9%	5.8% 6.5%	
Divergent/absent	1.1%	9.5% 53.0%	
VAP^a^	18.0%	84.7% 40.5%	

a – genes that were detected in at least one of the 24 EHEC 2 strains, but not in all EHEC 2 strains.

**Table 3:** Percentages of Sakai genes found in tested strains sorted by serotype.

Serotype	Strain	Sakai genes on array *n *= 4,944	Backbone (shared with K-12) *n *= 3,696	Sakai-specific	
				
				phage-related *n *= 814	Bacterial *n *= 434
O26: [h11]	DEC 9f	78%	94%	30%	20%
O26:H11	DEC 10e	82%	96%	51%	22%
O26:H11	F5863	84%	97%	56%	25%
O26:H11	97–3250	85%	97%	65%	23%
O26: [h11]	413/89-1	83%	96%	56%	20%
O26: [h11]	DA-22	84%	97%	57%	24%
O26:H11	03-ST-296	84%	97%	54%	22%
O26:H11	CB 7505	84%	96%	59%	30%
O26:H11	*Average*	83%	96%	54%	23%
	*Stan. Dev.*^a^	2.2%	1%	10.3%	3.2%
O111: [h11]	DEC 8c	82%	94%	55%	19%
O111:H11	DEC 8d	77%	93%	31%	21%
O111: [h8]	C408	82%	95%	49%	24%
O111: [h8]	BCL71	83%	95%	58%	24%
O111: [h8]	ML178190	82%	95%	52%	23%
O111:H8	W29104	81%	95%	48%	23%
O111: [h8]	EK34	81%	95%	47%	24%
O111:H8	EK35	80%	94%	49%	23%
O111:H8	*Average*	82%	95%	51%	24%
	*Stan. Dev.*	1%	0.4	4%	0.5%
O118: [h16]	RW2030	84%	96%	58%	23%
O118: [h16]	RW1302	82%	95%	56%	19%
O118:H16	666/89	83%	95%	57%	21%
O118:H16	05482	82%	96%	53%	22%
O118:H16	EK36	83%	96%	53%	21%
O118:H16	EK37	84%	97%	55%	25%
O118:H16	*Average*	83%	96%	55%	22%
	*Stan. Dev.*	0.9%	0.8%	2.1%	2%
O153: [h11]	02–3751	84%	97%	60%	24%
O15: [h11]	RDEC-1	80%	94%	42%	22%
O55:H7	97–3256	84%	97%	33%	70%

a – Stan. Dev., standard deviation.

**Figure 2:**
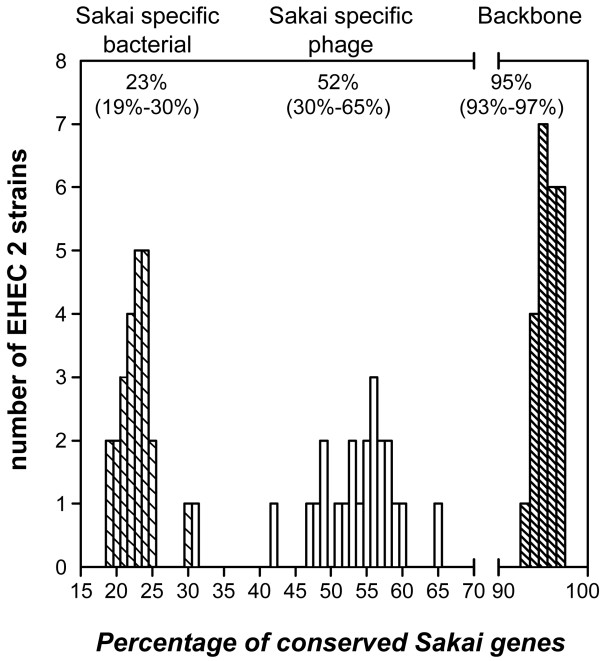
**Distribution of Sakai genes among EHEC 2 clinical strains**. The three histograms represent distribution trends of three Sakai gene groups in EHEC 2 strains: Sakai bacterial genes (left histogram – hatched bars), Sakai phage genes (middle histogram, open bars), and backbone genes (right histogram – hatched bars). The levels of Sakai gene content conservation were calculated for each EHEC 2 strain by dividing the number of Sakai genes, from a particular gene group, found in a strain by the total number of Sakai genes from the respective gene group, represented on the oligoarray; these values were expressed as percentages. Each bar represents the number of EHEC 2 strains that were found to have the same percentage of Sakai gene content conservation. Each strain is represented on each histogram and the bars in each histogram add up to 24, the total number of strains investigated. One exception is the bar representing Sakai phage gene content conservation in strain DEC9f, which is hidden by the hatched bar representing the Sakai bacterial gene content conservation in strain CB7505. As can be seen in Table 3, strain DEC9f has 30% of Sakai phage genes and strain CB7505 has 30% of Sakai bacterial genes, causing the bars to overlap. Numbers above each plot represent the average for each group of genes and the range of the distribution is given in parentheses.

In each individual EHEC 2 strain, approximately 95% of the 3,696 backbone genes were found (Table 3, Figure 2), with little variation (95.5% ± 1.2%, range 93% – 97%). In contrast, about 52% of the Sakai phage genes were found, but with a much greater variability across EHEC 2 strains (52.1% ± 8.2%, range 30% – 65%). This may be an over estimation of Sakai phage gene distribution in EHEC 2, as 231 of the 814 phage gene probes analyzed had multiple phage gene targets in the Sakai genome, based on *in silico *analysis of probe specificity. Sakai bacterial genes were found less frequently in EHEC 2 strains (22.7% ± 2.3%, range 19% – 30%). Serotype O26:H11 showed the most interstrain variation, whereas O111:H8 and O118:H16 were more uniform with respect to Sakai gene distribution. The O55:H7 representative also had a high percentage of backbone genes (96.6%). Furthermore, 33% of the 814 Sakai phage genes and 70% of the 434 Sakai bacterial genes were conserved in O55:H7, suggesting an inverse trend relative to that observed in EHEC 2 strains (Table 3).

### Identification of potential EHEC-specific genes

From the 1,248 Sakai-specific genes represented on the microarray, 152 (12.2%) were conserved in 23 of the 24 EHEC 2 strains; 102 of these were phage-related. Sixty-four genes encode hypothetical proteins of unknown function, and the remainder consisted mostly of genes responsible for various prophage and other mobile element functions. Nucleotide sequences of these 152 genes were compared against five non-EHEC pathogenic *E. coli *(536, APEC O1, B171, CFT073, UTI89) and six *Shigella *(Sf2a 2457T, Sf2a 301, Sf5 8401, Ss046, Sb227, Sd197) published genomes, using BLAST. With a minimum of 80% nucleotide sequence identity in a minimum of 80% query coverage as the cutoff value to identify conserved genes, 26 of the 152 genes were not found in any of the 11 queried non-EHEC genome sequences. The 26 gene sequences were then "BLASTed" against the entire GenBank database with the same cutoff value. Only three of these 26 genes were not found in any other organisms and therefore could be considered as specific to EHEC strains: ECs1561 (Sakai prophage (Sp) 6); ECs1763, and ECs1822 (Sp 9). All three genes encode hypothetical proteins of unknown function.

### Genomic relatedness of EHEC 2 strains

**Figure 3:**
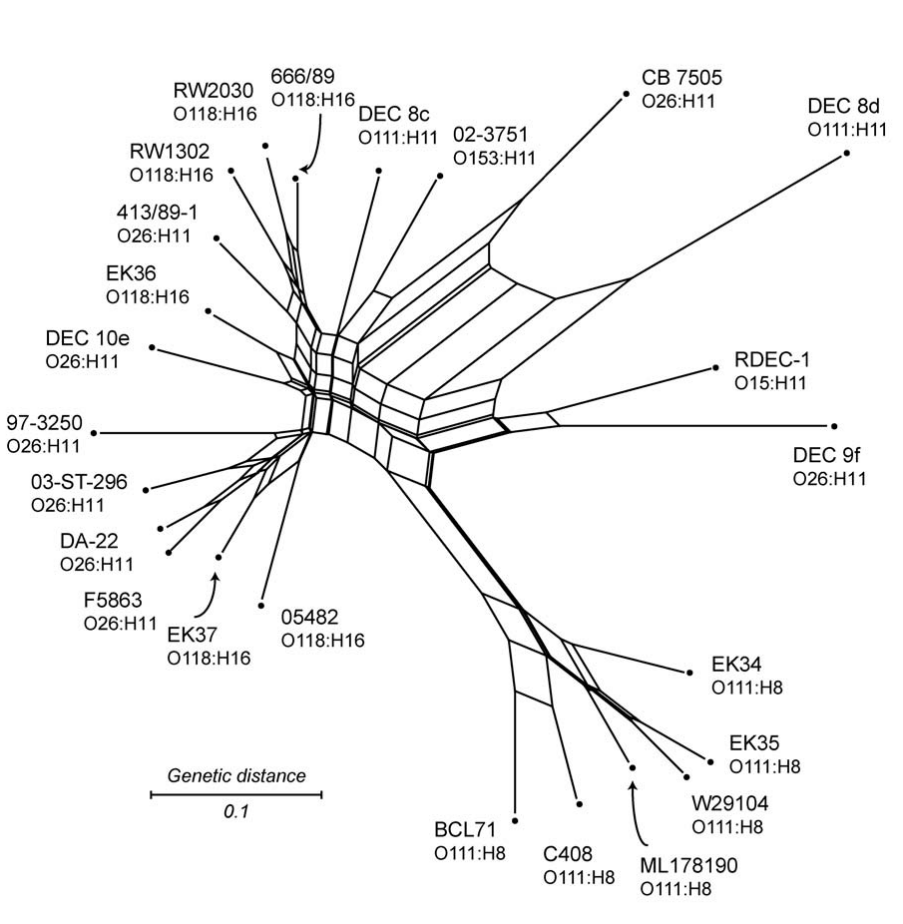
**Split decomposition analysis of Sakai genes in 24 EHEC 2 strains**. The network was generated based on the presence/absence of 4800 Sakai genes among 24 EHEC 2 strains. 144 genes were excluded because their probe intensities were below those of randomized negative controls in the various Sakai/EHEC 2 hybridizations. Node labels refer to strain names (listed in Table 1). Parallel edges represent phylogenetic incompatibilities in the data set, which are indicative of parallel gene gain/loss by multiple transduction events. The network was generated in Splitstree 4.3, using neighbor net with the uncorrected *p *distance. Scale bar represents number of gene differences (present or divergent/absent) per gene site.

We used the split decomposition method to infer the strain relatedness based on gene content data. We first analyzed all the 4,800 genes whose probe intensities were higher than those for negative controls. As expected, the analysis showed a network like phylogeny (Figure 3), in which the parallel edges reflected incompatible signals in the data that were indicative of parallel gene gain/loss due to multiple transduction events or past recombination. All O111:H8 strains were clustered closely and branched away from the remaining EHEC 2 strains, which formed a loose cluster without any recognizable concordance to serotypes, hosts, or locations (Figure 3). The pairwise homoplasy index (PHI) [43], generated in Splitstree, confirmed that there was significant evidence of recombination (*p*-value = 0.0).

**Figure 4:**
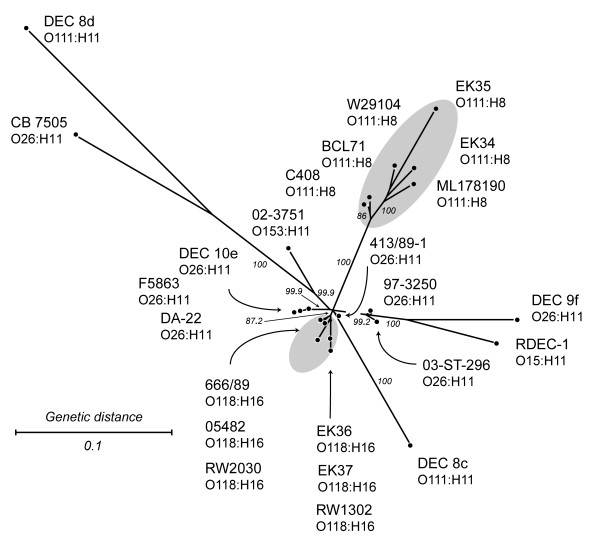
**Split decomposition analysis of compatible parsimony informative genes and singleton genes in 24 EHEC 2 strains**. Gray ovals encompass serotype-specific clusters of O118:H16 and O111:H8 strains. Node labels refer to strain names (listed in Table 1). The network was generated in Splitstree 4.3, using neighbor net with the uncorrected *p *distance. Scale bar represents number of gene differences (present or divergent/absent) per gene site. Percent bootstrap confidence values based on 1000 replicates are shown for selected edges.

Among the 4,800 genes whose probe intensities were higher than those for negative controls, 70.8% were found to be either present or divergent/absent in all 24 strains, and therefore, phylogenetically uninformative. Compatibility analysis of the 979 parsimoniously informative (PI) genes identified 147 PI genes to be phylogenetically compatible with each other, but not compatible with the rest of the PI genes (the distribution of these genes is shown in Additional file 1). For the second split decomposition analysis, these 147 genes were combined with 421 singleton genes (genes found present or divergent/absent in only one of the 24 EHEC 2 strains). Singletons were added to generate terminal edges of the network and to help distinguish strain-specific changes. The analysis with this set of genes showed a more tree like phylogeny with a better separation of EHEC 2 strains (Figure 4). Six O111:H8 strains and six O118:H16 strains formed two tight and distinct clusters, while the twelve O26:H11, O111:H11, O153:H11, and O15:H11 strains were dispersed throughout the network. The O111:H8 cluster was visibly distinct from the rest, reiterating its particular pattern of gene content conservation across all 4,800 genes (Figure 3). The two O111:H11 strains did not cluster with O111:H8 strains, which is not unusual since the O111 serogroup has been suggested to include several lineages [44]. In this analysis, the O118:H16 strains appear to be more closely related to most of the O26:H11 strains than any other EHEC 2 serotype. Nonetheless, there was a short edge separating the O118:H16 serotype from O26:H11, followed by strain-specific splits within O118:H16 that were based on singleton genes. The eight O26:H11 strains did not cluster together, suggesting that strains of this serotype are considerably more diverse than O111:H8 and O118:H16 strains.

### Prophages

**Figure 5:**
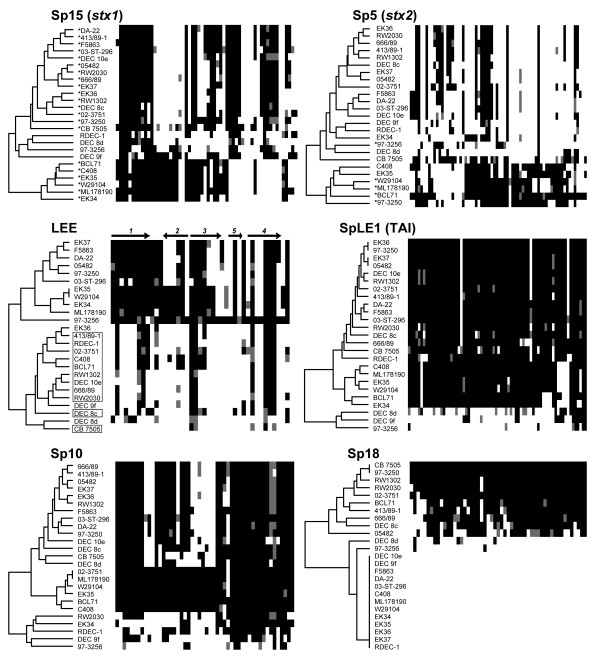
**Gene content of Sakai phage genes and the LEE island in EHEC 2 strains**. Sakai phage genes inferred as present or divergent/absent were grouped and sorted according to the Sakai annotation. Colormaps, with dendrograms, of individual phages were generated in R software (v 2.4.0.), using the 'gplots' package (v 2.3.2). Present genes are depicted as black, absent/divergent as white. Gray squares symbolize genes that have been classified as present after the cutoff was relaxed for 20%, representing a 'low' level of gene divergence. Dendrogram labels refer to strain names (Table 1). Labels with asterisks in the Sp15 and Sp5 colormaps refer to strains that were positive for *stx1 *and *stx2 *genes, respectively. Labels with open boxes in the LEE colormap represent animal strains. Arrows and numerals atop the LEE colormap represent operons and the direction of their transcription. The ECs numbers for the phage genes depicted, and the distribution of these genes, are provided in Additional file 2. Sp – Sakai prophage, SpLE – Sakai prophage-like element, TAI – tellurite resistance and adherence island.

To visualize gene content of the 814 Sakai phage genes within the EHEC 2 clonal group, we classified these genes by Sakai phage groups (Sakai prophages Sp1–18, and prophage-like elements SpLE1–6) and sorted the genes in each group by chromosomal order (based on ECs numbers). This classification does not necessarily infer that these genes are present in EHEC 2 within the same phage or order as they are in Sakai, but simply allows an assessment of gene content variation of laterally acquired genes known to be linked in the Sakai chromosome. Dendrograms based on pairwise comparison of gene content were used to identify EHEC 2 strains with similar gene content (Figure 5 and Additional file 2). Overall, there was no common pattern of gene distribution for all phage groups (Figure 5), which was also implied by additional split decomposition networks (data not shown). Some similarity was detected among O111:H8 strains for Sp5, Sp15 and Sp8 genes, with more Sp5 and Sp15 genes being conserved in the O111:H8 serotype than in other EHEC 2 strains. Conversely, Sp8 was well-conserved in all but the O111:H8 strains (data not shown), in which Sp8 genes were virtually absent except for two short gene segments, ECs1638–43 and ECs1656–63, which encode tail and hypothetical proteins, respectively.

### Stx converting prophages

The CGH data confirmed the *stx1*/*stx2 *profile of the EHEC 2 strains determined by PCR. In Sp15 (*stx1*-prophage), a block of genes at the beginning of the phage (ECs2940–2952) was conserved in most strains (Figure 5). These genes encode tail proteins and the putative outer membrane protein Lom precursor (ECs2942). Adjacent is a group of genes (ECs2953–2963) encoding two tail proteins, a putative terminase large subunit and several unknown proteins, which are fully conserved in O111:H8 strains but almost completely divergent/absent in the rest. Two regions in the Sp15 phage, ECs2984–2988 and ECs2998–3006, were well conserved in all strains positive for the *stx1 *gene, except in O111:H8 strains. Excisionase and integrase genes (ECs3012 and ECs3013) were divergent/absent in most of the EHEC 2 strains. Overall, the gene content of Sp15 in strains negative for the *stx1 *gene was different from those in *stx1 *positive strains (Figure 5).

Strains positive for the *stx2 *gene, mostly representing serotype O111:H8, had more Sp5 (*stx2*-phage) genes. Integrase and excisionase genes (ECs1160 and ECs1161), and the block of genes at the beginning of the phage, ECs1160–1187, were missing from most strains. The rest of Sp5 genes, which encode replication proteins O and P, NinE and NinG, Shiga toxin 2, antirepressor proteins, antitermination protein Q, outer membrane precursor proteins, terminases, tail proteins, and a number of hypothetical proteins, were present in five of the six O111:H8 strains as well as in the O26:H11 strain containing both *stx1 *and *stx2 *(Figure 5).

### Locus of enterocyte effacement (LEE) island

Of the 41 genes in the Sakai LEE island that are located on SpLE4, all except *escU *were present in the O55:H7 strain. This includes genes that were categorized as present after the initial GACK cutoff was relaxed by 20%. Since dye-swap genomic microarrays represent competitive hybridizations between two populations of DNA, there were instances when a small difference in the nucleotide sequence of the tested strain resulted in weaker probe signal intensity. For example, both of the two known SNPs present between the variable regions of γ intimin in O55:H7 and O157:H7 [45] are located in the middle region of the 70-mer probe for *eae*. Hence the signal intensity for this gene was just below the cutoff (gray shading in Figure 5). Based on the level of divergence of EHEC 2 LEE genes from O157 LEE genes, strains clustered into two major groups (Figure 5). The top group of the dendrogram is composed of human strains, which have a high level of similarity to O157 LEE genes, whereas the bottom cluster represents 11 animal and 3 human strains that have a lower level of similarity to the O157 LEE genes. The level of divergence was also found to be heterogeneous between LEE operons (Table 4). The genes that encode the type III secretion system (TTSS), *escRSTUCJVNDF*, were detected in 14 to 24 strains, with the exception of *escR *and *escC*, which were found in 11 and 5 strains, respectively. The needle filament gene, *espA*, was present in 23 strains, whereas *espB *and *espD *were divergent/absent in all. The *tir *and γ intimin genes were also divergent/absent in EHEC 2; the γ intimin was conserved only in the O55:H7 representative, an expected result because the 70-mer probe was designed to detect the variable (allele-specific) part of *eae*.

**Table 4:** Conservation of O157 LEE operons in a set of 24 EHEC 2 strains.

	LEE1 (9)^a^	LEE2 (6)	LEE3 (7)	LEE5 (3)	LEE4 (8)
Human^b^	8.8 ± 0.6	3.1 ± 1.7	4.8 ± 1.1	1.0 ± 0.0	4.4 ± 0.5
Animal^c^	3.2 ± 1.2	0.6 ± 0.9	2.7 ± 0.9	0.1 ± 0.2	3.8 ± 0.9

a. The number of genes in each operon is given in parentheses.

b. Refers to the 10 human isolates in the top cluster of the dendrogram in the LEE image in Figure 5, not including O55:H7. Values represent average number of genes in an operon, with standard deviation.

c. Refers to the 11 animal and 3 human isolates in the bottom cluster of the dendrogram in the LEE image in Figure 5. Values represent average number of genes in an operon, with standard deviation.

### Other phage gene groups

Most genes from SpLE1, which encodes the tellurite resistance and adherence island (TAI), were divergent/absent from two EHEC 2 strains and from the O55:H7 representative, but present in the rest of the EHEC 2 strains (Figure 5). The diverse trend in retention or loss of laterally acquired genes was emphasized by the arrangement of Sp10 genes. CGH data inferred three patterns of Sp10 gene content conservation in EHEC 2 (Figure 5). In the first 14 strains (top to bottom), Sp10 genes were found to be present or divergent/absent in an *en bloc *fashion. The middle branch of the dendrogram represents six strains in which virtually all Sp10 genes were present. In the remaining five strains, Sp10 genes appeared to have a mosaic structure with individual genes present or divergent/absent. In contrast, Sp18 was either entirely divergent/absent or nearly completely present. There was no correlation between the distribution of Sakai phage genes in EHEC 2 and geographic location of the EHEC 2 isolates.

### Non-LEE encoded effectors

The gene content of non-LEE encoded effectors, which are predicted to be secreted by the LEE-encoded TTSS [42] in EHEC 2, varied from totally divergent/absent to present in every strain (Additional file 3). Genes *espY1*, *nleD*, *espX2*, *espY4*, *espL3'*, *espX3'*, *espL4*, and *nleB2-1 *were divergent/absent from EHEC 2, whereas a set of 15 genes (*espX1*, *espX5*, *espX6*, *espY3*, *espK*, *nleA*, *nleE*, *nleG*, *nleG2-2*, *nleG6-1*, *espM1*, *espM2*, *espR1*, *espL1*, and *espW*) were present in at least 22 EHEC 2 strains. The *nleG7 *gene, which was recently found to be conserved in a group of non-O157 EHEC strains [46], was also divergent/absent in all EHEC 2 examined in this study.

## Discussion

Comparative analysis of genomes from 17 commensal and pathogenic *E. coli *strains has revealed a diverse species 'pan-genome', while the *E. coli *'core conserved' genome was calculated to be about one-half of the genome of a given *E. coli *isolate [47]. Although EHEC utilize similar virulence mechanisms, this pathotype is comprised of phylogenetically distinct lineages that vary in their ability to cause disease in both humans and animals. Clearly, the genome of a single strain cannot reflect how the genomic diversity among EHEC strains influences pathogenesis of the EHEC population. Because no strains from the EHEC 2 clonal group have been sequenced, the genetic variability of 24 EHEC 2 strains were examined in relation to the distribution of genes from O157:H7 Sakai, which belongs to the EHEC 1 clonal group. The Sakai genome was used in this study, as its annotation is suggested to include more strain-specific genes compared to EDL933 [47]. Genes specific to the EHEC 2 group have yet to be described. Some genes shared with Sakai might have been missed in our study, if the gene sequence had diverged to a point where the 70-mer oligonucleotide probes and the stringency of competitive hybridization preclude detection. Although this study allowed screening of known genes only, the gene content data still offered new insight on strain relatedness and the distribution and subsequent diversification of mobile elements within the EHEC 2 clonal group.

The CGH data presented here indicate that there are two distinct trends, which reflect the bacterial (vertical) and phage (lateral) origin of genes, impacting the genomic divergence of EHEC 2. Virtually the entire set of backbone genes was present within the EHEC 2 clonal group (Tables 2 and 3). CGH inferences pertaining to the distribution of backbone genes can vary depending on array type, sample size, and strain diversity [46]. For example, Anjum *et al*. have proposed that the O26 serogroup exhibits greater genetic homogeneity than was observed in our study [48]; however, the microarray platform used in that study was limited to the genome of K-12 MG1655. Despite these differences, the degree of conservation among backbone genes in this CGH investigation was similar in previous studies [46,49,50]. The distribution of Sakai-specific genes in EHEC 2 was, not surprisingly, noticeably lower than that of the backbone, which restates established findings about intraspecies genomic variability [40,51,52]. The conservation of Sakai phage genes was, however, found to be more than 2-fold higher when compared to Sakai bacterial genes (Figure 2 and Table 3). In O55:H7, the inferred ancestor of O157:H7 [53], the proportion of Sakai phage to bacterial gene conservation was opposite from the proportion observed in EHEC 2; this suggests that Sakai bacterial genes have been vertically acquired from the O55:H7 progenitor and are not disseminated among the EHEC 2 clone. Cursory assessment of K-12-specific genes suggests a homogenous distribution in EHEC 2, with less than half of the genes present; most K-12 phage-related genes were found to be uniformly divergent/absent from the entire EHEC 2 population (Additional file 4). Assessing the conservation of K-12 specific genes was, however, beyond the scope of this study, as K-12 MG1655 is a non-pathogenic laboratory-derived strain that is distantly related to EHEC (Figure 1).

The increased presence of Sakai phage genes in the EHEC 2 group compared to Sakai bacterial genes reveals independent acquisition and exchange of similar mobile elements. For example, of the 152 Sakai-specific genes present in EHEC 2, only 26 genes were not found in 11 completed non-EHEC *E. coli *and *Shigella spp*. genomes. About one-half of the 26 "EHEC only" genes were found in *stx1*-encoding phages BP-4795 and CP-1639 from STEC O84:H11 and O111:H-, respectively [54,55]. Sakai genes identified by BLASTN as present on BP-4795 are disseminated on phages Sp6, 9, 10, and 12, which is in agreement with the evidence for recombination between phages [56]. Although the number of phage genes shared by all tested strains was low, the percentage of those that were VAP was high (Table 2), which may reflect sequence heterogeneity in prophage genomes with similar modular structures [54,56,57], and not true absence of genes.

Phylogenetic network analysis implied a serotype-specific uniformity of O111:H8 strains, unlike other EHEC 2 strains (Figure 3), which can also be inferred from the arrangement of Sakai phage genes in O111:H8 strains (Figure 5). Interestingly, these six EHEC 2 representatives are the only strains with the θ intimin allele while the remaining eighteen EHEC 2 strains had β intimin, as determined by PCR-based RFLP typing of *eae*; the method for *eae *typing was described previously [58]. By contrast, members of the EHEC 1 clonal group (i.e., O157:H7 and O55:H7) typically had the γ allele. Although intimin θ has been found in an atypical EPEC O55:H7 and a non-EHEC 2 strain (GenBank Acc. No. AJ833638 and AF253561), O111:H8 is, to our knowledge, the only EHEC 2 serotype with this intimin allele, providing further support for the hypothesis that O111:H8 represents a distinct grouping.

Based on the distinguishing distribution of Sakai genes (Figures 3 and 4), serotype O26:H11 appears to be considerably more diverse compared to the distinct and more uniform O111:H8. This suggests that the genetic make-up of O26:H11 is such that it allows more frequent lateral exchange of DNA elements, which can result in acquisition of novel fitness and virulence genes by O26:H11 more commonly than by other EHEC 2. For example, O26:H11 possess the *Yersinia spp*. high pathogenicity island (HPI) that encodes the iron-uptake siderophore yersiniabactin and the pesticin receptor, whereas other EHEC serotypes, including O157:H7, O111:H-, O103:H2, and O145:H-, do not have this HPI [59]. The diversity of O26:H11/H- has also been implied with other methods [60].

A proportion of the EHEC 2 hybridization data (15% of the PI genes) were identified as genes that are phylogenetically compatible with each other, i.e., having no homoplasy. Although this represents a small number of genes, it is remarkable that the distribution pattern grouped EHEC 2 O111:H8 and O118:H16 strains by serotype (Figure 4). The pathogenic *E. coli *used in this study represent tips of phylogenetic branches, where high frequencies of recombination strongly impact the shaping of genomic content [61] and eventually lead to erosion of the phylogenetic signal between clonal complexes [62]. Thus, the set of genes shared with EHEC 1 O157:H7 whose pattern of presence and absence in EHEC 2 infers compatibility and is not random, but coincides with serotype, warrants further investigation.

The heterogeneity of Stx phages has been demonstrated [57,63], even within the O157:H7 lineage itself [64,65], so it is not unexpected to find such variation between different EHEC 2 strains. In addition, Ogura *et al*. propose that Stx phages have alternative integration sites in EHEC 2 [46]; this may explain our lack of detection of integrase genes, as integration site specificity is dependent on the alignment of the phage integrase with the attachment sequence in the bacterial chromosome [66]. Strains that were *stx *negative in our study were, nevertheless, found to carry genes from the Sp15 and Sp5 phages, which is a common effect of frequent modular shuffling of sequences between phages of related enteric hosts [56,67,68]. The significance of the unique conservation patterns of Sp10 and Sp18 phage genes is not clear. Sp10 is perhaps more conserved as it harbors non-LEE effector genes [42], all 3 of which were detected in at least 22 out of 24 EHEC 2 strains. Absence of the entire Sp18 was also detected among O157:H7 strains [65], one of which belongs to a hyper-virulent lineage of the O157:H7 population [69].

Incongruent divergence of LEE operons has been previously suggested. Studies indicate that this island is a dynamic region [70], and that different selective pressures act on different parts of the LEE [71]. The sequence diversity of the LEE, both at the nucleotide and amino acid level, increases along the length of the island from the LEE1 to the LEE4 operon [71,72]. A comparable trend can be observed in the CGH data presented here, as there was greater conservation of the content of genes that encode the secretion apparatus (LEE1–3). However, differences in the content of O157:H7 Sakai LEE genes between human and animal EHEC 2 strains of the same serotype (Figure 5 and Table 4) suggest that the LEE has diverged between EHEC 2 strains in a host dependent manner, possibly due to host species adaptive pressure. This result was not expected and its implications are not supported by the current literature. Multiple, parallel acquisitions of the LEE by different clonal groups have been inferred [37,73-75].

Muniesa *et al*. suggest that the LEE genes associated with serogroup O26 are present more commonly in STEC than the LEE genes associated with EHEC O157:H7 or EPEC O127:H6 [76]. Yet, there is no clear evidence to support the hypothesis that LEE divergence within a lineage results from positive adaptive pressure in different host species. In fact, when several LEE genes from strain RDEC-1 were compared to those from other AEEC, the variation appeared to be associated with evolutionary lineage and not host specificity [77]. Even so, given the heterogeneous diversification of this island and the recent inference about host-specific expression of *espA *and *eae *in O157:H7 [78], it would be interesting to compare complete LEE sequences from a larger sample of EHEC 2 strains of human and animal origin.

## Conclusion

Here, we present an assessment of the gene content of a set of EHEC 2 clinical strains of animal and human origin, isolated from the USA and Europe. The small subset of phylogenetically compatible genes represent potential markers that will aid in the investigation of the relatedness and cladogenesis of the EHEC 2 clonal group. In this study, serotype O26:H11, the most frequent EHEC 2 serotype associated with overt disease, represented the most diverse EHEC 2 population. Compared to the more homogeneous O111:H8 strains, O26:H11 strains may have an increased propensity to laterally exchange DNA, which may ultimately give rise to hyper-virulent lineages within EHEC 2 O26:H11. Furthermore, the identification of several EHEC-specific genes could potentially be used as novel genetic markers to identify strains belonging to this pathotype.

## Methods

### Bacterial strains and DNA isolation

Since genome sequences for tested strains are not available, two-color hybridizations between sequenced strains of *E. coli *O157:H7 RIMD 0509952 (Sakai) [38] and K-12 MG1655 [79] were used as references. A total of 24 EHEC 2 strains including serotypes O26:H11 (*n *= 8), O111:H8 (*n *= 6), O111:H11 (*n *= 2), O118:H16 (*n *= 6), O153:H- (*n *= 1), and O15:H11 (*n *= 1), originally isolated from human and animal cases of STEC-associated disease, were used in this study and were selected based on the serotype and source (Table 1) [6,33,80-90]. The study also included an EHEC 1 O55:H7 strain, isolated from a human diarrhea case. Bacterial DNA was prepared from overnight LB cultures grown at 37°C using the Puregene genomic DNA isolation kit (Gentra Systems, Minneapolis, MN).

### Multilocus sequence typing (MLST) and Shiga toxin (Stx) genes

The detailed MLST protocol and multiplex PCR conditions for characterizing the Stx genes (*stx1*/*stx2*) can be found at the STEC Reference Center website . Briefly, MLST was performed on seven conserved housekeeping genes (*aspC*, *clpX*, *fadD*, *icdA*, *lysP*, *mdh*, and *uidA*), and sequence type (ST) assignments were made based on phylogenetic analyses of the concatenated sequences.

### Oligonucleotide arrays

The Qiagen (Valencia, Calif.) spotted multi-genome arrays containing probes specific for 5,978 ORFs from *E. coli *K-12 MG1655, O157:H7 Sakai and EDL933 were utilized. Of these probes, a total of 5,943 were 70-mer oligonucleotides and 35 ranged from 41–69 bp. The probes were printed in duplicate on UltraGaps glass slides (Corning Inc., NY) at the Research Technology Support Facility at Michigan State University. The array also contained 384 spots representing 12 randomized negative control 70-mer probes. All probes were assigned ORF designations (b- = MG1655, ECs- = Sakai, or Z- = EDL933 numbers) or intergenic region labels based on the RefSeq database available on the National Center for Biotechnology Information (NCBI) website [91].

### *In silico *analysis of microarray probe specificity

To verify the probes with the up-to-date genome annotations, we compared all 5,990 probe sequences against the three *E*. *coli *genomes (MG1655, Sakai, and EDL933) by BLASTN available on NCBI, and recorded the two highest hits for every probe (top hit and second hit) for each genome. A probe was considered to be specific for a target when the top hit demonstrated ≥ 80% identity to the probe sequence stretch in the strain. Probes with nonspecific hybridization and multiple target hybridizations within MG1655 or Sakai DNA were excluded from the data analysis of MG1655 and Sakai hybridizations. These included probes that had multiple top hits with 75% overall identity or probes that had multiple top hits between 50% and 75% of overall identity with alignments containing a stretch of nucleotides with 100% identity, in which the stretch was 20% of the probe length. With respect to the MG1655 and Sakai genomes, out of 5,978 probes, 12 had no target (EDL933 specific), 731 showed nonspecific hybridization or had multiple targets, and 5,235 matched single genome targets. Of these, 3,803 targeted both genomes, with 1,002 targeting only Sakai and 430 targeting only K-12.

### DNA labeling and microarray hybridization

Genomic DNA was sheared into 500 to 5,000 bp fragments in a cup sonicator (Heat Systems Ultrasonics W-225, 20 KHz, 200 W) and 250 ng of sheared DNA was labeled with aminoallyl-dUTP (Sigma, St. Louis, Mo.) using the Invitrogen (Carlsbad, Calif.) DNA labeling system, as previously described [40]. Equal amounts of DNA from Sakai and test strains were suspended and combined in a final volume of 44 μL of SlydeHyb Buffer #1 (Ambion, Inc., Austin, TX). Qiagen *E. coli *spotted oligoarrays were hybridized and washed according to the manufacturer's instructions for hybridization using coverslips. Test strains were hybridized twice with Sakai as a reference: once with the Cy5 labeled test strain and Cy3 labeled Sakai and once with the Cy3 labeled test strain and Cy5 labeled Sakai to correct for dye incorporation bias.

### Data collection and analyses

Arrays were scanned with the Genepix 4000B array scanner (Axon Instruments, Union City, Calif.) and probe intensities (median pixel intensities) were retrieved using Genepix 6.0 (Axon Instruments). Data quality was assessed by viewing plots of *M *versus *A *[*M *= log_2 _(test/reference); *A *= log_2 _(test × reference)] and by checking for spatial effects with Genepix 6.0 and GeneTraffic (Iobion, La Jolla, Calif.) as described previously [40]. Because genome sequences of tested strains were not available, microarray data were not normalized to avoid biasing the gene content of tested strains. Instead, microarray images showing spatial bias were discarded and hybridizations were repeated until control parameters were appropriate. Duplicate probes for each gene were averaged prior to analyses. Probes with median pixel intensities higher than the median of the randomized negative controls were analyzed as the distribution of the two-color signal ratios using the "GACK" program [92]. Analysis of the log_2 _(test strain/reference strain) distribution (GACK_1_) as well as of the reciprocal ratio, log_2 _(reference strain/test strain) (GACK_2_), were performed for Sakai versus MG1655 hybridizations to determine a cutoff. Genes with a GACK_1 _value of ≥ 0.1 were classified as present, whereas genes with a GACK_1 _value of < 0.1 were classified as divergent/absent. At this cutoff, maximum sensitivity (98.8%) and specificity (96%) were achieved for the MG1655/Sakai dye-swap hybridizations, and therefore, this cutoff was used to interpret the data from Sakai versus EHEC 2 hybridizations. The term 'present' is used to indicate that a gene was detected by CGH, and does not necessarily imply that the whole gene is conserved or functional; likewise, the term 'divergent/absent' indicates that a gene was not detected by CGH.

### Phylogenetic analyses

Strains were assigned to clonal groups based on STs and bootstrap analyses as described previously [36,93]. A neighbor-joining tree of the concatenated MLST sequences was constructed using the Kimura 2-parameter distance method with 1000 bootstrap replications in MEGA 3.1 [94]. The tree includes other enteropathogenic *E. coli *(EPEC) and EHEC STs as well as the lab-derived K-12 (ST173) and the uropathogenic *E. coli *CFT073 (ST27) for comparison; an *E. albertii *strain was used as the outgroup. For phylogenetic analyses of the microarray data, a total of 144 genes (from all array hybridizations) with probe intensities below those of negative controls were excluded from the set of 4,944 genes. Neighbor-net phylogenies highlighting the distribution of Sakai genes in EHEC 2 strains, for which the presence or absence of genes was coded as 0 (divergent/absent) or 1 (present), were constructed using the uncorrected *p *distance in Splitstree 4.3 [95]. The number of Sakai genes whose distribution in EHEC 2 was parsimoniously informative were determined in MEGA 3.1 [94], and the set of Sakai genes in EHEC 2 whose distribution was compatible with a single phylogeny was identified using the clique module of PHYLIP [96].

## Competing interests

The authors declare that they have no competing interests.

## Authors' contributions

GSA designed the study, collected and analyzed CGH data, and drafted the manuscript. DWL performed intimin and *stx *typing, phylogenetic analyses and helped to draft the manuscript. LMW participated in design and analysis of CGH data and WQ performed *in silico *verification of microarray probe specificity. TSW participated in the design and coordination of the study, conducted the phylogenetic analyses, and helped draft the manuscript. The first four authors read and approved the final manuscript; TSW had approved an earlier draft of the manuscript (deceased, December 5, 2008).

## Supplementary Material

Additional file 1 **Distribution of phylogenetically compatible genes in EHEC 2, determined with the clique program in the PHYLIP package**. Conserved genes have a value of 1 and divergent/absent have a value of 0. Click here for file

Additional file 2 **Genes in EHEC 2 whose distribution was used to generate colormaps in Figure **5. Conserved genes have a value of 1, divergent/absent genes have a value of 0 and genes that have a value of 0.5 were inferred as conserved after the GACK cutoff was relaxed by 20%. Click here for file

Additional file 3 **Distribution of 49 non-LEE effector genes in EHEC 2**. Conserved genes have a value of 1, divergent/absent genes have a value of 0 and genes that have a value of 0.5 were inferred as conserved after the GACK cutoff was relaxed by 20%. Click here for file

Additional file 4 **Distribution of K-12-specific genes in EHEC 2**. Conserved genes have a value of 1 and divergent/absent genes have a value of 0. Click here for file
